# A mixed-modeling framework for analyzing multitask whole-brain network data

**DOI:** 10.1162/netn_a_00065

**Published:** 2019-02-01

**Authors:** Sean L. Simpson, Mohsen Bahrami, Paul J. Laurienti

**Affiliations:** Department of Biostatistical Sciences, Wake Forest School of Medicine, Winston-Salem, NC, USA; Laboratory for Complex Brain Networks, Wake Forest School of Medicine, Winston-Salem, NC, USA; Laboratory for Complex Brain Networks, Wake Forest School of Medicine, Winston-Salem, NC, USA; Department of Biomedical Engineering, Wake Forest School of Medicine, Winston-Salem, NC, USA; Laboratory for Complex Brain Networks, Wake Forest School of Medicine, Winston-Salem, NC, USA; Department of Radiology, Wake Forest School of Medicine, Winston-Salem, NC, USA

**Keywords:** Graph theory, Connectivity, fMRI, Small-world, Network mixed model, Longitudinal

## Abstract

The emerging area of brain network analysis considers the brain as a system, providing profound insight into links between system-level properties and health outcomes. Network science has facilitated these analyses and our understanding of how the brain is organized. While network science has catalyzed a paradigmatic shift in neuroscience, methods for statistically analyzing networks have lagged behind. To address this for cross-sectional network data, we developed a mixed-modeling framework that enables quantifying the relationship between phenotype and connectivity patterns, predicting connectivity structure based on phenotype, simulating networks to gain a better understanding of topological variability, and thresholding individual networks leveraging group information. Here we extend this comprehensive approach to enable studying system-level brain properties across multiple tasks. We focus on rest-to-task network changes, but this extension is equally applicable to the assessment of network changes for any repeated task paradigm. Our approach allows (a) assessing population network differences in changes between tasks, and how these changes relate to health outcomes; (b) assessing individual variability in network differences in changes between tasks, and how this variability relates to health outcomes; and (c) deriving more accurate and precise estimates of the relationships between phenotype and health outcomes within a given task.

## INTRODUCTION

Human brain imaging, particularly physiological methods like functional magnetic resonance imaging (fMRI) (Ogawa, Lee, Kay, & Tank, 1990), has revolutionized our understanding of the human brain. Currently we are experiencing another revolution in brain imaging—the application of network science. Network theory applied to neuroscience has endorsed new ways of viewing brain organization and has led to new insights into complex emergent brain function. These functional brain network analyses have moved to the forefront of neuroimaging research and serve as a distinct subfield of functional connectivity analysis (FC) (Biswal, Yetkin, Haughton, & Hyde, 1995; Friston, 1994; Simpson & Laurienti, 2016; Sporns, 2010) in which functional associations are quantified for all *n* parcellated time series pairs to create an interconnected representation of the brain (a brain network). The resulting *n* × *n* connection matrix is often thresholded to remove negative connections (for reasons noted in Cao et al., 2014; Telesford, Simpson, Burdette, Hayasaka, & Laurienti, 2011; and others) and/or weak connections.

Despite the fact that network science has catalyzed a paradigmatic shift in neuroscience, the current statistical tools and toolboxes used to analyze this network data have lagged behind, failing to fully harness the wealth of information present and provide the flexibility of the modeling and inferential tools developed for fMRI activation data (Simpson, Bowman, & Laurienti, 2013; Simpson & Laurienti, 2015). The systemic organization present in brain networks confers much of our brains’ functional abilities. Functional connections may be lost because of an adverse health condition, but compensatory connections may develop to maintain organizational consistency and functional abilities. Consequently, brain network analysis necessitates a suite of tools including a multivariate modeling framework to assess the effects of multiple variables of interest and topological network features on the overall network structure. That is, if we have

$$\text{Data}\left\{\begin{array}{l} Y_{i}:\text{network}\;\text{of}\;\text{participant}\;i\;\\ X_{i}:\text{covariate}\;\text{information}\;\text{(metrics,}\;\text{demographics,}\;\text{etc.)}\; \end{array}\right.\text{,}$$

we want the ability to model the probability density function of the network given the covariates *P*(***Y***_*i*_|***X***_*i*_, ***θ***_*i*_), where ***θ***_*i*_ are the parameters that relate the covariates to the network structure. The multivariate distance matrix regression (MDMR) framework provides a relatively recent, powerful addition to this suite of analysis tools (Shehzad et al., 2014). This framework allows controlling for confounding covariates in group comparisons via a “pseudo-*F*” statistic. However, it lacks the ability to simulate networks or make predictions as it doesn’t directly model *P*(***Y***_*i*_|***X***_*i*_, ***θ***_*i*_). It also fails to account for the dependence in connectivity patterns across regions.

**Figure 1.**
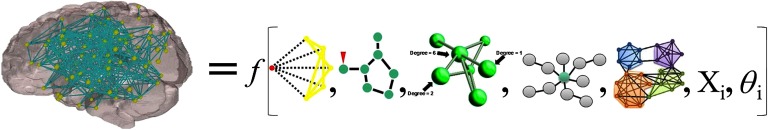
Modeling a brain network as a function of endogenous (network measures: clustering, global efficiency, degree, centrality, and modularity illustrated respectively) and exogenous (***X***_*i*_) variables of interest (age, gender, disease status, etc.). ***θ***_*i*_ represents parameter estimates and model errors.

To address these needs and further add to the tool suite, we developed a two-part mixed-modeling framework to enable multivariate analyses of connectivity and its relationship to endogenous variables (that summarize network topology) and exogenous variables (those of biological relevance that may be associated with changes in network topology) (Figure 1) (Bahrami et al., 2017; Simpson & Laurienti, 2015), as well as a corresponding user-friendly toolbox (Bahrami, Laurienti, & Simpson, 2018). More specifically, this framework directly models *P*(***Y***_*i*_|***X***_*i*_, ***θ***_*i*_), and enables quantifying the relationship between phenotype and connectivity patterns in the brain after controlling for confounders, predicting connectivity structure based on phenotype, simulating networks to gain a better understanding of normal ranges of topological variability, and thresholding individual networks leveraging group information for a given task. To our knowledge, no comparable alternative frameworks currently exist. However, our mixed-modeling-approach has not yet been developed for multitask network data. Univariate comparisons of metrics across tasks dominate in multitask analyses (Ginestet, Fournel, & Simmons, 2014), precluding the assessment of the relationship between population network differences and individual variability in network differences in between-task changes and health outcomes. Importantly, the link between rest-to-task brain network changes and health outcomes, particularly in aging, is underexplored because of the lack of appropriate analytic tools. Here we extend our comprehensive mixed-modeling approach to enable studying system-level brain properties across multiple tasks. We focus on rest-to-task network changes, but this extension is equally applicable to the assessment of network changes for any repeated task paradigm, including interrelated task designs such as those employed in multisensory studies. Our approach allows (a) assessing population network differences in changes between tasks, and how these changes relate to health outcomes (i.e., drawing direct inference about task-related differences in phenotype-network organization relationships); (b) assessing individual variability in network differences in changes between tasks, and how this variability relates to health outcomes; and (c) deriving more accurate and precise estimates of the relationship between phenotype and health outcomes within a given task by leveraging information from other tasks.

For the following discussion of the mixed-modeling framework for multitask brain network data, we describe the motivating data concerning age-related cognitive decline in the next section. We then detail our modeling approach and its utility, and use the aging data to illustrate the use of the proposed framework. We conclude with a summary discussion including planned future research.

## MATERIALS AND METHODS

### Motivating Example

Our data come from a prior study that aimed to assess the neurological underpinnings of age-related cognitive decline by examining the effects of aging on the integration of sensory information (Hugenschmidt, Mozolic, Tan, Kraft, & Laurienti, 2009). The study protocol was approved by the Wake Forest School of Medicine Institutional Review Board. All subjects gave written informed consent in accordance with the Declaration of Helsinki. The study has two age groups, healthy young adults aged 27 ± 5.8 years old (*n* = 20) and healthy older adults aged 73 ± 6.6 years old (*n* = 19). Three separate conditions of fMRI scans were used, resting, visual (viewing of a silent movie), and multisensory (MS) (visual and auditory—movie with sound), each lasting 5.6 min. Further details about these conditions along with additional network analyses can be found in a previous publication (Moussa et al., 2011). For each fMRI scan, blood-oxygen-level dependence (BOLD) contrast was measured using a 1.5T MRI scanner and a whole-brain gradient echo-planar imaging (EPI) sequence with the following parameters: 200 volumes with 24 contiguous slices per volume; slice thickness = 5.0 mm; in-plane resolution of 3.75 mm × 3.75 mm; TR = 3,000 ms.

To process the data, functional scans were normalized to standard brain space with a 4 × 4 × 5 mm voxel size. Data were band pass filtered (0.00765–0.068 Hz), and motion parameters, global signal, and mean white matter (WM) and cerebral spinal fluid (CSF) signals were regressed from the imaging time series data. Brain networks for each participant were then constructed by calculating Pearson correlation coefficients between these denoised motion-corrected time courses for all node pairs (see Hayasaka & Laurienti, 2010, for further details). These node time courses were obtained by averaging the voxel time courses in the 90 distinct anatomical regions (90 ROIs—regions of interest) defined by the Automated Anatomical Labeling atlas (AAL; Tzourio-Mazoyer et al., 2002). The resulting 90 × 90 connection matrices were then thresholded to remove negative connections (for reasons noted in Cao et al., 2014; Telesford et al., 2011; and others) resulting in sparse weighted networks.

We previously analyzed these data with our two-part mixed-modeling framework for cross-sectional network data to compare the network topology between the 20 young and 19 older adults *within* the three task states (rest, visual, and multisensory), fitting a separate model for each task (Simpson & Laurienti, 2015). Here we extend the framework to enable the examination of rest-to-task network topology changes between the groups. Although these data are relatively old and have been evaluated in our prior study, performing analyses on these same data allows clearly distinguishing their utility and exemplifying the differences in inference, and thus the differences in conclusions, that *can* occur by fitting the more appropriate multitask model to multitask data.

### Mixed-Modeling Framework for Multitask Weighted Brain Networks

#### Definition.

Given that we have sparse weighted networks, a two-part mixed-effects model will be employed to model both the probability of a connection (presence/absence) and the strength of a connection if it exists (Simpson & Laurienti, 2015). The model includes the entire brain connectivity matrix of each participant, endogenous covariates, and exogenous covariates (see Figure 1). The endogenous covariates are summary variables extracted from the network to summarize global topology. The exogenous covariates are the biologically relevant phenotypic variables (e.g., for our data, sex, educational attainment, and age). This statistical framework allows for the evaluation of group and individual effects. Another key feature of the model is that it allows accounting for the dependencies among edges in a network given its multivariate statistical formulation, thus increasing the power to detect differences and decreasing false positives (Edwards, 2000). The inclusion of the actual connectivity matrices allows the statistics to be performed on the entire network simultaneously rather than performing edge-by-edge analyses in a massively univariate fashion.

Specifically, let *Y*_*ijkl*_ represent the *strength* of the connection (quantified as the correlation in our case) and *R*_*ijkl*_ indicate whether a connection is present (*presence* variable) between node *j* and node *k* for the *i*th subject during the *l*th task. Thus, *R*_*ijkl*_ = 0 if *Y*_*ijkl*_ = 0, and *R*_*ijkl*_ = 1 if *Y*_*ijkl*_ > 0 with conditional probabilities

$$P\left(R_{ijkl}=r_{ijkl}|\beta_{rl};b_{rli}\right)=\left\{\begin{array}{ll} 1-p_{ijkl}\left(\beta_{rl};b_{rli}\right)\; & \text{if}\;r_{ijkl}=0\\ p_{ijkl}\left(\beta_{rl};b_{rli}\right)\; & \text{if}\;r_{ijkl}=1 \end{array}\right.\text{,}$$ (1)

where ***β***_*rl*_ is a vector of population parameters (fixed effects) that relate the probability of a connection to a set of covariates (***X***_*ijkl*_) for each subject and nodal pair (dyad) for the *l*th task, and ***b***_*rli*_ is a vector of subject- and node-specific parameters (random effects) that capture how this relationship varies about the population average (***β***_*rl*_) by subject and node (***Z***_*ijkl*_) for the *l*th task. Hence, *p*_*ijkl*_ (***β***_*rl*_; ***b***_*rli*_) is the probability of a connection between nodes *j* and *k* for subject *i* during the *l*th task. We then have the following logistic mixed model (part I model) for the probability of this connection:

$$logit\left(p_{ijkl}\left(\beta_{rl};b_{rli}\right)\right)=X_{ijkl}^{\prime }\beta_{rl}+{\text{Z}}_{ijkl}^{\prime }b_{rli}.$$ (2)

For the part II model, which aims to model the strength of a connection given that there is one, we let *S*_*ijkl*_ = [*Y*_*ijkl*_|*R*_*ijkl*_ = 1]. In our case, the *S*_*ijkl*_ will be the values of the correlation coefficients between nodes *j* and *k* for subject *i* during the *l*th task. We can then use Fisher’s Z-transform, denoted as *FZT*, and assume normality for the following mixed model (part II model):

$$FZT\left(S_{ijkl}\left(\beta_{sl};b_{sli}\right)\right)=X_{ijkl}^{\prime }\beta_{sl}+{\text{Z}}_{ijkl}^{\prime }b_{sli}+e_{ijkl}\text{,}$$ (3)

where ***β***_*sl*_ is a vector of population parameters that relate the strength of a connection to the same set of covariates (***X***_*ijkl*_) for each subject and nodal pair (dyad) for the *l*th task, ***b***_*sli*_ is a vector of subject- and node-specific parameters that capture how this relationship varies about the population average (***β***_*sl*_) by subject and node (***Z***_*ijkl*_) for the *l*th task, and *e*_*ijkl*_ accounts for the random noise in the connection strength of nodes *j* and *k* for subject *i* during the *l*th task.

The covariates (***X***_*ijkl*_) used to explain and predict both the presence and the strength of connection are (a) *Net*: the average of the following network measures (categorized in Table 1 and further detailed in Rubinov & Sporns, 2010, and Simpson et al., 2013) in each dyad: clustering (*C*), global efficiency (*Eglob*), degree (*k*) (difference instead of average to capture “assortativity”), modularity (*Q*), and leverage centrality (*l*); (b) *COI*: covariates of interest (age group in our case); (c) *Int*: interactions of the covariate of interest with the metrics in (a); and (d) *Con*: confounders (sex, years of education, (Euclidean) spatial distance (between nodes) [importance of geometric distance noted by Friedman, Landsberg, Owen, Li, & Mukherjee, 2014], and the square of spatial distance in our case). Thus, we can decompose ***β***_*rl*_ and ***β***_*sl*_ into ***β***_*rl*_ = [***β***_*rl*,0_ ***β***_*rl*,*net*_ ***β***_*rl*,*coi*_ ***β***_*rl*,*int*_ ***β***_*rl*,*con*_]′ and ***β***_*sl*_ = [***β***_*sl*,0_ ***β***_*sl*,*net*_ ***β***_*sl*,*coi*_ ***β***_*sl*,*int*_ ***β***_*sl*,*con*_]′ to correspond with the population intercepts and these covariates (see the Parameter interpretations subsection below for further details on parameter interpretations). For the random-effects vectors we have that ***b***_*rli*_ = [***b***_*rli*,0_ ***b***_*rli*,*net*_ ***b***_*rli*,*dist*_ ***δ***_*rli*,*j*_ ***δ***_*rli*,*k*_]′ and ***b***_*sli*_ = [***b***_*sli*,0_ ***b***_*sli*,*net*_ ***b***_*sli*,*dist*_ ***δ***_*sli*,*j*_ ***δ***_*sli*,*k*_]′, where ***b***_*rli*,0_ and ***b***_*sli*,0_ quantify the deviation of subject-specific intercepts from the population intercepts (***β***_*rl*,0_ and ***β***_*sl*,0_), ***b***_*rli*,*net*_ and ***b***_*sli*,*net*_ contain the subject-specific parameters that capture how much the relationships between the network measures in (a) and the *presence* and *strength* of a connection vary about the population relationships (***β***_*rl*,*net*_ and ***β***_*sl*,*net*_) respectively, ***b***_*rli*,*dist*_ and ***b***_*sli*,*dist*_ contain the subject-specific parameters that capture how much the relationship between spatial distance and the *presence* and *strength* of a connection vary about the population relationships respectively, ***δ***_*rli*,*j*_ and ***δ***_*sli*,*j*_ contain nodal-specific parameters that represent the propensity for node *j* (of the given dyad) to be connected and the magnitude of its connections respectively, and ***δ***_*rli*,*k*_ and ***δ***_*sli*,*k*_ contain nodal-specific parameters that represent the propensity for node *k* (of the given dyad) to be connected and the magnitude of its connections respectively. Parameters for all *t* tasks (*l* = 1, 2, …, *t*) are estimated or predicted simultaneously from the model. In general, additional exogenous covariates can also be incorporated as guided by the biological context with standard covariate selection procedures (e.g., backward selection) employed as needed. We recommend using all five endogenous covariates (Table 1) to cover the major categories of systemic network properties and account for confounding given that the metrics are mildly correlated. However, a strong scientific rationale may justify fitting fewer (or more) than the five along with the removal (or addition) of the corresponding endogenous random effects.

**Table 1.** Explanatory network metrics by category

**Category**	**Metric(s)**
1) Functional segregation	Clustering coefficient

2) Functional integration	Global efficiency

3) Resilience	Degree difference

4) Centrality and information flow	Leverage centrality

5) Community structure	Modularity

Specifying a reasonable covariance model (balancing appropriate complexity with parsimony and computational feasibility) is paramount for a unified multitask model such as the one developed here. Toward this end, we assume that ***b***_*ri*_ = [***b***_*r*1*i*_ ***b***_*r*2*i*_ … ***b***_*rti*_]′, ***b***_*si*_ = [***b***_*s*1*i*_ ***b***_*s*2*i*_ … ***b***_*sti*_]′, and ***e***_*i*_ = {***e***_*ijk*_ = [*e*_*ijk*1_ *e*_*ijk*2_ … *e*_*ijkt*_]′} are normally distributed and mutually independent, with variance component covariance structures for ***b***_*rli*_ and ***b***_*sli*_ for each group (age group in our case), and the standard conditional independence structure for ***e***_*i*_. That is, ***b***_*rli*_ ∼ *N* (**0**, **Σ**_*rli*_ (***τ***_*rl*_) = diag(***τ***_*rl*_)) where

$$\tau_{rl}={\left(\sigma_{rl,0}^{2},\;\sigma_{rl,net}^{2},\;\sigma_{rl,dist}^{2},\;\sigma_{rl,node1}^{2},\;\sigma_{rl,node2}^{2},\;\ldots ,\;\sigma_{rl,node90}^{2}\right)}^{\prime }$$

and ***b***_*sli*_ ∼ *N* (**0**, **Σ**_*sli*_ (***τ***_*sl*_) = diag(***τ***_*sl*_)) where

$$\tau_{sl}={\left(\sigma_{sl,0}^{2},\;\sigma_{sl,net}^{2},\;\sigma_{sl,dist}^{2},\;\sigma_{sl,node1}^{2},\;\sigma_{sl,node2}^{2},\;\ldots ,\;\sigma_{sl,node90}^{2}\right)}^{\prime }$$

are the 98 × 1 vectors of variances for each element of the random-effects vectors for each group, and ***e***_*i*_ ∼ *N* (**0**, **Σ**_*ei*_ = *σ*^2^***I***). Additionally, the model contains covariance parameters for each random effect and its counterparts across tasks for each group yielding 98 × t2 covariance parameters for both the *presence* and *strength* models per group. That is, the overall random-effects covariance is modeled with unstructured covariance matrices (parameterized through their Cholesky roots) for each random effect and its counterparts across tasks. For example, in the two-task, one-group case we have that

$$Cov\left(b_{rl_{1}i},b_{rl_{2}i}\right)=\left(\begin{array}{cc} \Sigma_{rl_{1}i} & \;D_{rl_{1}l_{2}i}\\ \cdots & \;\Sigma_{rl_{2}i} \end{array}\right),$$

where ***D***_*rl*_1_*l*_2_*i*_ = diag(***λ***_*r*_), and

$$Cov\left(b_{sl_{1}i},b_{sl_{2}i}\right)=\left(\begin{array}{cc} \Sigma_{sl_{1}i} & \;D_{sl_{1}l_{2}i}\\ \cdots & \;\Sigma_{sl_{2}i} \end{array}\right),$$

where ***D***_*sl*_1_*l*_2_*i*_ = diag(***λ***_*s*_).

These covariance parameters, contained in the ***λ***_*r*_ and ***λ***_*s*_ vectors for the example, provide insight into whether individual and group differences in between-task variability relate to health and behavioral outcomes. Parameter estimation is conducted via restricted pseudo-likelihood (Wolfinger & O’Connell, 1993) with the residual approximation of the *F* test for a Wald statistic employed for inference.

This modeling framework allows *explaining* the relationship between covariates and network connectivity across tasks, *comparing* network connectivity among groups and across tasks, *predicting* network connectivity based on participant and nodal characteristics and task state, *simulating* networks as a means of assessing goodness-of-fit and better understanding network topological variability, and *thresholding* networks leveraging group and across-task information. These analyses allow drawing direct inference about task-related differences in phenotype-network organization relationships, providing further understanding of the brain changes that occur during task changes and transitions. These differences provide complementary insight to within-task analyses. As with all biological systems, studying the brain at various levels (micro, meso, macro) remains paramount, especially given the hierarchical nature of its physiology. This requires analyzing connectivity properties (specific interregional connections) and higher-level network properties (systemic architecture). Our approach is a hybrid method that allows this multilevel assessment.

#### Parameter interpretations.

Specific interpretations of fixed-effect parameters (after centering continuous covariates) for our data context are given below.

*β*_*rl*,0_ and *β*_*sl*,0_ – The log odds of an edge existing and the average strength of that connection for dyads with average values for the network metrics and spatial distance from the network of a young male with the average educational attainment for the *l*th task.
***β***_*rl*,*net*_ and ***β***_*sl*,*net*_ – The change in the log odds of an edge existing and the average strength of that connection for a dyad with each unit increase in the given network metric from the networks of young adults for the *l*th task.
*β*_*rl*,*age*_ and *β*_*sl*,*age*_ – The change in the log odds of an edge existing and the average strength of that connection for dyads from the networks of older males with average values for the network metrics for the *l*th task.
*β*_*rl*,*sex*_ and *β*_*sl*,*sex*_ – The change in the log odds of an edge existing and the average strength of that connection for dyads from the networks of younger females for the *l*th task.
*β*_*rl*,*educ*_ and *β*_*sl*,*educ*_ – The change in the log odds of edges existing and the average strength of those connections with each year increase in educational attainment for the *l*th task.
*β*_*rl*,*dist*_/*β*_*r*,*dist*^2^_ and *β*_*sl*,*dist*_/*β*_*s*,*dist*^2^_ – The quadratic change in the log odds of an edge existing and the average strength of that connection with each millimeter (scaled to decimeter for model fit) increase in spatial distance between the two nodes of a given dyad for the *l*th task.
***β***_*rl*,*age*×*net*_ and ***β***_*sl*,*age*×*net*_ – The additional change (relative to ***β***_*rl*,*net*_ and ***β***_*sl*,*net*_) in the log odds of an edge existing and the average strength of that connection for a dyad with each unit increase in the given network metric from the networks of older adults for the *l*th task.
*β*_*rl*,*age*×*sex*_ and *β*_*sl*,*age*×*sex*_ – The additional change (relative to *β*_*rl*,*sex*_ and *β*_*sl*,*sex*_) in the log odds of an edge existing and the average strength of that connection for dyads from the networks of older females for the *l*th task.

#### Additional and unique framework utilities.

*1. More accurate and precise within-task results* Our unified multitask model enables more accurate and precise estimation of the relationship between phenotype and health outcomes within a given task since it is able to leverage information from other tasks, particularly through the covariance parameters that capture correlations across tasks. This improvement in within-task accuracy and precision (and general model robustness) over the unitask (cross-sectional) approach is an inherent benefit of accounting for the dependencies among multiple observations (networks in our case) from the same individual (Edwards, 2000). Moreover, simulating within-task brain networks from the unitask and multitask mixed models, and assessing how well the observed networks match the simulated networks, provides the most appropriate way to compare the goodness-of-fit of both models in the network context (Hunter, Goodreau, & Handcock, 2008). We show that the multitask mixed-modeling framework provides a better fit to the data in this way, and thus, we can be more confident in the results it yields.
*2. Assess population network differences and individual variability in network differences within and between tasks* Our framework allows assessing how group membership, and phenotypic and demographic characteristics, relate to how the brain changes between tasks (an assessment that cannot be made with a cross-sectional approach) via estimation of [(***β***_*rl*,0_ − ***β***_*rl*′,0_) + (***β***_*rl*,*net*_ − ***β***_*rl*′,*net*_)] and [(***β***_*sl*,0_ − ***β***_*sl*′,0_) + (***β***_*sl*,*net*_ − ***β***_*sl*′,*net*_)] for each population/group of interest or as a continuous function of a phenotypic/demographic characteristic of interest. This assessment is important for many scientific domains, but particularly for aging since the underlying mechanism causing cognitive decline is likely related to how the brain changes from one task to another, and not just to the network structure during a given task. Our framework also allows assessing how group membership, and phenotypic and demographic characteristics, affect the ability of the brain to change smoothly between tasks via estimation of Var [(***β***_*rl*,0_ − ***β***_*rl*′,0_) + (***β***_*rl*,*net*_ − ***β***_*rl*′,*net*_)] and Var [(***β***_*sl*,0_ − ***β***_*sl*′,0_) + (***β***_*sl*,*net*_ − ***β***_*sl*′,*net*_)] for each population/group of interest or as a continuous function of a phenotypic/demographic trait of interest. Unstable, highly variable changes could be indicative of dysfunction.

Our framework also allows assessing how a specific individual’s (participant *i*) brain differs between tasks (again, an assessment that cannot be made with a cross-sectional approach) via prediction of [((***β***_*rl*,0_ + ***b***_*rli*,0_) − (***β***_*rl*′,0_ + ***b***_*rl*′,*i*,0_)) + ((***β***_*rl*,*net*_ + ***b***_*rli*,*net*_) − (***β***_*rl*′,*net*_ + ***b***_*rl*′,*i*,*net*_))] and [((***β***_*sl*,0_ + ***b***_*sli*,0_) − (***β***_*sl*′,0_ + ***b***_*sl*′,*i*,0_)) + ((***β***_*sl*,*net*_ + ***b***_*sli*,*net*_) − (***β***_*sl*′,*net*_ + ***b***_*sl*′,*i*,*net*_))] given their phenotypic and demographic characteristics. It also enables illuminating how much the changes of individuals tend to deviate from population changes via examination of $\sigma_{rl,0}^{2}$, $\sigma_{rl,net}^{2}$, $\sigma_{sl,0}^{2}$, and $\sigma_{sl,net}^{2}$. Additionally, the 98 × t2 covariance parametersper group for both the *presence* and *strength* models provide insight into group differences in between-task dynamics.

## RESULTS

**Figure 2.**
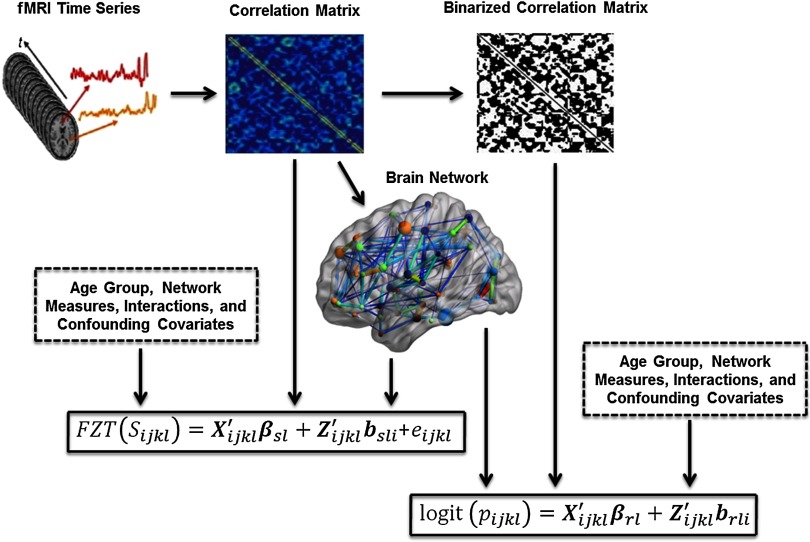
Diagram of the modeling approach. fMRI data were collected from each study participant. The average time series was determined from 90 anatomical brain regions as defined in the AAL atlas. Each region served as a network node. The correlation matrix was obtained by calculating the Pearson correlation between the average time series from every node pair, with negative correlations set to 0. The binarized correlation matrix was obtained by setting all nonzero correlations from the correlation matrix to 1. The network measures were extracted from the correlation matrix. These metrics along with age group, the interactions of age group with the network measures, and the noted confounding variables were used as covariates in the two-part mixed-effects modeling framework for multitask data.

As noted earlier, we previously analyzed the aging study data with a unitask mixed modeling framework and illustrated its utility in comparing the network topology between 20 young and 19 older adults within three task states (rest, visual, and multisensory), fitting a separate model for each task (Simpson & Laurienti, 2015). Here we reanalyzed these data with our multitask mixed-modeling framework, allowing the additional and unique assessments delineated in the previous section and illustrated in this section. We fitted one model to the rest and visual data together and one to the rest and multisensory data together given a lack of convergence when attempting to fit all three together. The model-fitting process for each pair of tasks consisted of first estimating all 902 edges for each of the 39 subjects and two tasks (i.e., 902 × 39 × 2 total edge estimates) by calculating Pearson correlation coefficients between the time courses of all nodal pairs as detailed in the second paragraph of the Motivating example subsection. Then the multitask mixed model was fitted to all 78 (39 subjects × 2 tasks) of these vectorized adjacency matrices of length 902 to relate them to the noted covariates in the Definition subsection (age group, network measures, interactions, confounding variables: sex, years of education, [Euclidean] spatial distance, and square of spatial distance [between nodes]). That is, the outcome variables for the model are the vectorized adjacency matrices for each subject and task. This approach is diagramed in Figure 2 (partially recreated from Bahrami et al., 2017, and Xia, Wang, & He, 2013). Future work will examine data-reduction approaches to facilitate convergence with larger numbers of tasks. All standard mixed-modeling assumptions and diagnostics were checked (Cheng, Edwards, Maldonado-Molina, Komro, & Muller, 2010; Muller & Fetterman, 2002). We provide the essential SAS macro for model fitting in Supplementary Appendix S1 (Simpson, Bahrami, & Laurienti, 2019). Supplementary Appendices S2 and S3 from Simpson & Laurienti, 2015, can be modified accordingly to create prediction intervals and conduct simulations for this multitask context.

*1. More accurate and precise within-task results* As noted previously, our unified multitask model enables more accurate and precise estimation of the relationship between phenotype and health outcomes within a given task than the unitask (cross-sectional) approach since it is able to leverage information from other tasks, particularly through the covariance parameters that capture correlations across tasks (Edwards, 2000). Additionally, the multitask model provides a better fit to the data in the network context (i.e., enables simulating more realistic brain networks; Hunter et al., 2008) as evidenced by the simulation results in Table 2. Results in this table came from first simulating 100 networks based on separate unitask model fits and our new joint rest/visual and rest/multisensory multitask data fits. To simulate each network we first simulated the existence of edges for all 4,005 node pairs from a Bernoulli distribution with the probability from the fitted model (*p*_*ijkl*_ (***β***_*rl*_; ***b***_*rli*_) from Equation 2) using mean values for the covariates and the appropriate indicator variables to indicate the task. To get simulated strength values, we then multiplied the resulting binary vector by simulated values from a normal distribution with parameters from the corresponding fitted model from Equation 3 $\left(N\left(X_{ijkl}^{\prime }\beta_{sl},Z_{ijkl}\Sigma_{sli}\left(\tau_{sl}\right){\text{Z}}_{ijkl}^{\prime }+\sigma^{2}I\right)\right)$ using mean values for the covariates and the appropriate indicator variables to indicate the task, and subsequently used the inverse Fisher’s Z-transform to get the untransformed values. We then calculated several (weighted) descriptive metrics commonly used in the network neuroscience literature for the observed and simulated networks: clustering coefficient (*C*), global efficiency (*Eglob*), characteristic path length (*L*), mean nodal degree (*K*), leverage centrality (*l*), and modularity (*Q*). Our new approach better captures the topological properties of the observed networks, and thus provides a better fit to the data, as evidenced by the fact that the average Euclidean distance between these properties for the simulated networks and the observed networks for unitask models is 0.5901, whereas the average distance for multitask models is 0.3087, a 48% improvement.

**Table 2.** Weighted network metrics of observed and simulated networks from the aging study data produced from the original univariate mixed-model fits and new multivariate fits

**Condition**	**Metric**	**Observed (*N* = 39)**	**Simulated (*N* = 100) [Mean (SE)]**		
		**Mean (*SE*)**	**Univar**	**Rest/Visual**	**Rest/MS**
Rest	Clustering coefficient (*C*)	0.149 (0.001)	0.155 (0.000)	0.124 (0.000)	0.132 (0.000)
	Global efficiency (*Eglob*)	0.231 (0.001)	0.214 (0.000)	0.237 (0.000)	0.250 (0.000)
	Characteristic path length (*L*)	4.677 (0.109)	4.720 (0.004)	4.274 (0.006)	4.080 (0.005)
	Mean nodal degree (*K*)	10.649 (0.055)	12.747 (0.016)	9.723 (0.029)	10.321 (0.026)
	Leverage centrality (*l*)	2.678 (0.015)	1.945 (0.003)	2.103 (0.005)	2.025 (0.004)
	Modularity (*Q*)	0.342 (0.001)	0.136 (0.000)	0.218 (0.000)	0.188 (0.000)

Visual	Clustering coefficient (*C*)	0.150 (0.001)	0.150 (0.000)	0.131 (0.000)	NA
	Global efficiency (*Eglob*)	0.232 (0.001)	0.205 (0.000)	0.245 (0.000)	NA
	Characteristic path length (*L*)	4.553 (0.073)	4.992 (0.008)	4.161 (0.005)	NA
	Mean nodal degree (*K*)	10.656 (0.056)	12.379 (0.025)	9.884 (0.025)	NA
	Leverage centrality (*l*)	2.671 (0.015)	2.155 (0.010)	2.021 (0.004)	NA
	Modularity (*Q*)	0.348 (0.001)	0.136 (0.000)	0.194 (0.000)	NA

Multisensory	Clustering coefficient (*C*)	0.140 (0.001)	0.165 (0.000)	NA	0.132 (0.000)
	Global efficiency (*Eglob*)	0.230 (0.001)	0.218 (0.000)	NA	0.250 (0.000)
	Characteristic path length (*L*)	4.431 (0.011)	4.700 (0.008)	NA	4.080 (0.005)
	Mean nodal degree (*K*)	10.547 (0.052)	13.830 (0.027)	NA	10.321 (0.026)
	Leverage centrality (*l*)	2.862 (0.014)	2.054 (0.006)	NA	2.025 (0.004)
	Modularity (*Q*)	0.327 (0.001)	0.123 (0.000)	NA	0.188 (0.000)

Given the inherent benefits of jointly modeling multiple tasks and accounting for the correlations between them, as well as the improved goodness-of-fit of our approach in the network context, we can be more confident in the results it yields than those of the unitask approach. Differences in the parameter estimates, standard errors, and *p* values between the previous unitask mixed-model fit (“Rest (univariate)” columns of Table 3 and Supplementary Table 1; Simpson et al., 2019) and the multitask model fit (“Rest (with visual)” and “Rest (with multisensory)” columns of Table 3 and Supplementary Table 1; Simpson et al., 2019) exemplify the differences in inference, and thus the differences in conclusions, that *can* occur by fitting the more appropriate multitask model to multitask data. Values that are bolded and italicized indicate substantial differences in the estimated relationships between covariates and network connectivity at rest (that we deemed notable) resulting from the leveraging of task information in the model. For the sake of brevity we highlight those differences resulting from the rest/visual multitask fit below, with a parallel detailing of the rest/multisensory results in the Supporting Information section (Simpson et al., 2019). Also of note, the standard errors from both multitask model fits are generally (but not uniformly) smaller than those from the unitask model fit.

**Table 3.** Aging data: Estimates, standard errors (*SE*), and *p* values for the original univariate mixed-model fit to rest data and new multivariate fit to rest (and visual) data

**Parameter *l*_1_ = rest**	**Rest (univariate)**			**Rest (with visual)^a^**		
	**Estimate**	***SE***	*p* value*	**Estimate**	***SE***	*p* value*
*β*_*rl*_1_,0_	−0.3141	0.0569	<0.0001	−0.2488	0.0448	<0.0001
*β*_*rl*_1_,*C*_	0.7807	0.3424	0.0355	***7.1274***	1.8466	***0.0004***
*β*_*rl*_1_,*EGlob*_	32.6231	2.3322	<0.0001	30.6695	1.3489	<0.0001
*β*_*rl*_1_,*k*_	−1.4442	0.1522	<0.0001	−1.5229	0.1782	<0.0001
*β*_*rl*_1_,*Q*_	−0.7345	1.1361	0.5179	−0.6460	0.9376	***0.4923***
*β*_*rl*_1_,*l*_	1.1598	0.0785	<0.0001	1.3824	0.0907	<0.0001
*β*_*rl*_1_,*age*_	−0.0438	0.0773	0.5709	−0.0806	0.0792	0.4229
*β*_*rl*_1_,*sex*_	−0.0085	0.0825	0.9178	−0.0452	0.0658	***0.4923***
*β*_*rl*_1_,*educ*_	0.0027	0.0103	0.7954	0.0071	0.0097	0.4923
*β*_*rl*_1_,*dist*_	−1.4266	0.0572	<0.0001	−1.4648	0.0530	<0.0001
*β*_*rl*_1_,*dist*^2^_	2.6558	0.1417	<0.0001	2.6309	0.1062	<0.0001
*β*_*rl*_1_,*age*×*C*_	1.1249	0.7986	0.1943	***−2.7472***	2.2936	0.3429
*β*_*rl*_1_,*age*×*EGlob*_	−1.7255	3.3478	0.6063	−0.3192	2.5953	0.9021
*β*_*rl*_1_,*age*×*k*_	0.2455	0.2185	0.2873	0.2682	0.2270	0.3430
*β*_*rl*_1_,*age*×*Q*_	1.5858	1.5753	0.3141	0.6680	1.5041	0.6570
*β*_*rl*_1_,*age*×*l*_	0.0638	0.1154	0.5803	***−0.0844***	0.1449	0.5604
*β*_*rl*_1_,*age*×*sex*_	0.1914	0.1145	0.1301	0.1999	0.1179	0.1802

*β*_*sl*_1_,0_	0.2290	0.0091	<0.0001	0.2253	0.0108	<0.0001
*β*_*sl*_1_,*C*_	2.2940	0.2428	<0.0001	2.2476	0.1614	<0.0001
*β*_*sl*_1_,*EGlob*_	0.9534	0.1823	<0.0001	1.0693	0.1869	<0.0001
*β*_*sl*_1_,*k*_	−0.2524	0.0153	<0.0001	−0.2438	0.0126	<0.0001
*β*_*sl*_1_,*Q*_	−0.0373	0.1723	0.8285	0.0364	0.1593	0.8195
*β*_*sl*_1_,*l*_	−0.0036	0.0109	0.7426	−0.0014	0.0115	0.9021
*β*_*sl*_1_,*age*_	−0.0106	0.0124	0.4262	−0.0080	0.0126	0.5228
*β*_*sl*_1_,*sex*_	−0.0046	0.0132	0.7263	0.0002	0.0151	0.9872
*β*_*sl*_1_,*educ*_	−0.0009	0.0017	0.5814	−0.0026	0.0015	***0.0933***
*β*_*sl*_1_,*dist*_	−0.1718	***0.0054***	<0.0001	−0.1717	0.0052	<0.0001
*β*_*sl*_1_,*dist*^2^_	0.3910	0.0118	<0.0001	0.3865	0.0117	<0.0001
*β*_*sl*_1_,*age*×*C*_	0.7214	0.3494	0.0467	0.5293	0.2508	0.0493
*β*_*sl*_1_,*age*×*EGlob*_	0.7042	0.2623	0.0097	0.6452	0.2269	0.0084
*β*_*sl*_1_,*age*×*k*_	0.0009	0.0220	0.9662	−0.0054	0.0208	0.7957
*β*_*sl*_1_,*age*×*Q*_	−0.6957	0.2464	0.0071	−0.6588	0.1996	0.0021
*β*_*sl*_1_,*age*×*l*_	0.0556	0.0157	0.0007	0.0464	0.0139	0.0019
*β*_*sl*_1_,*age*×*sex*_	0.0001	0.0184	0.9952	***−0.0083***	0.0181	0.6474

* Adjusted using the adaptive FDR procedure detailed in Benjamini and Hochberg (2000).

a Bold and italicized values indicate substantial differences in the estimated relationships between covariates and network connectivity at rest resulting from the leveraging of visual task information in the model.

1. Clustering coefficient (functional segregation/regional specificity) is shown to play an even greater role in explaining the connectivity (presence) between two regions at rest for both young and older adults as indicated by the change in the order of magnitude of *β*_*rl*_1_,*C*_, the increase in (*β*_*rl*_1_,*C*_ + *β*_*rl*_1_,*age*×*C*_), and the change in two orders of magnitude of the *p* value for *β*_*rl*_1_,*C*_. Additionally, the change in sign of *β*_*rl*_1_,*age*×*C*_ provides (weak) evidence that older adults have a weaker relationship between clustering and connectivity than young adults, whereas the opposite conclusion results from the unitask model fit.

2. While still not significant, there is stronger evidence for a relationship between sex and connection probability, education and connection probability, and sex and connection strength, as evidenced by the 46%, 38%, and 84% decreases in the corresponding *p* values.

3. The change in sign of *β*_*rl*_1_,*age*×*l*_ provides (weak) evidence that older adults have a weaker relationship between leverage centrality and connectivity than young adults, whereas the opposite conclusion results from the unitask model fit.

4. The change in sign of *β*_*sl*_1_,*age*×*k*_ provides (weak) evidence that the brain networks of older adults are actually more degree assortative (in terms of connection strength) than young adults at rest, not less as the unitask model indicates.

5. The change in sign of *β*_*sl*_1_,*age*×*sex*_ provides (weak) evidence that the brain networks of older females have weaker overall connection strength than young females, not stronger as the unitask model indicates.

*2. Assess population network differences and individual variability in network differences within and between tasks* Results from the fitted multitask models provided a comprehensive appraisal of across-task related network differences between the groups (again, an appraisal that cannot be made with the cross-sectional approach, or any current approach that we are aware of). As in the previous section, for the sake of brevity we detail the rest/visual results below, with a parallel detailing of the rest/multisensory results in the Supporting Information section (Simpson et al., 2019). The resulting estimated change in parameters, along with the standard errors and *p* values (based on the residual approximation of the *F* test for a Wald statistic) of the estimated change, associated with each of the fixed-effect covariates for the difference between the rest and visual task and the rest and multisensory task are presented in the last three columns of Table 4 and Supplementary Table 2 (Simpson et al., 2019) respectively. These estimates quantify the difference in the relationship between the endogenous network features and the probability and strength of a connection between nodes (brain areas), the difference in the relationship between age and the confounders (sex, years of education, spatial distance between regions, and the square of spatial distance) and the probability and strength of connections, and how the difference in the relationships between network features and the probability and strength of connections varies between young and older adults for rest and task states. Variance estimates for the random-effects parameters for the rest/visual and rest/multisensory fits are presented in Table 5 and Supplementary Table 3 (Simpson et al., 2019) respectively (estimates for the propensities and distance random effects not reported for the sake of brevity). These estimates quantify how much the brain networks of individuals within each group tend to deviate from their respective populations, and allow quantifying how much the rest-task differences of the brain networks of individuals within each group tend to deviate from the differences of their respective populations. Covariance parameter estimates are not reported given the minimal difference between groups and for the sake of brevity.

**Table 4.** Aging data (multivariate rest/visual fit): Estimates for visual and rest, and estimates, standard errors (*SE*), and *p* values for the between-task differences

**Parameter**	**Visual**	**Rest**	**Difference (visual − rest)^a^**		
	**Estimate**	**Estimate**	**Estimate**	***SE***	*p* value*
*β*_*r*,0_	−0.2897	−0.2488	−0.0409	0.0659	0.5351
*β*_*r*,*C*_	8.9255	7.1274	1.7981	2.4554	0.4923
*β*_*r*,*Eglob*_	35.9310	30.6695	5.2615	2.7262	0.1252
*β*_*r*,*k*_	−1.9930	−1.5229	−0.4701	0.2029	**0.0591**
*β*_*r*,*Q*_	−1.3026	−0.6460	−0.6566	1.8093	0.7167
*β*_*r*,*l*_	1.4900	1.3824	0.1076	0.1278	0.4722
*β*_*r*,*age*_	0.1655	−0.0806	0.2461	0.1005	**0.0467**
*β*_*r*,*sex*_	0.0408	−0.0452	0.0860	0.0907	0.4454
*β*_*r*,*educ*_	0.0023	0.0071	−0.0048	0.0128	0.7069
*β*_*r*,*dist*_	−1.5565	−1.4648	−0.0917	0.0603	0.2376
*β*_*r*,*dist*^2^_	2.6925	2.6309	0.0616	0.1684	0.7144
*β*_*r*,*age*×*C*_	−4.3221	−2.7472	−1.5749	2.8359	0.5786
*β*_*r*,*age*×*Eglob*_	−8.0698	−0.3192	−7.7506	4.0850	0.1252
*β*_*r*,*age*×*k*_	0.5917	0.2682	0.3235	0.2605	0.3430
*β*_*r*,*age*×*Q*_	4.7263	0.6680	4.0583	3.1713	0.3430
*β*_*r*,*age*×*l*_	−0.2372	−0.0844	−0.1528	0.1757	0.4722
*β*_*r*,*age*×*sex*_	−0.1383	0.1999	−0.3382	0.1597	0.0888

*β*_*s*,0_	0.2157	0.2253	−0.0096	0.0120	0.4231
*β*_*s*,*C*_	2.6607	2.2476	0.4131	0.2888	0.1525
*β*_*s*,*Eglob*_	1.4452	1.0693	0.3759	0.1996	**0.0676**
*β*_*s*,*k*_	−0.2828	−0.2438	−0.0390	0.0196	**0.0607**
*β*_*s*,*Q*_	−0.5634	0.0364	−0.5998	0.3306	**0.0740**
*β*_*s*,*l*_	0.0203	−0.0014	0.0217	0.0141	0.1226
*β*_*s*,*age*_	−0.0008	−0.0080	0.0072	0.0143	0.6136
*β*_*s*,*sex*_	0.0245	0.0002	0.0243	0.0163	0.1364
*β*_*s*,*educ*_	−0.0016	−0.0026	0.0010	0.0018	0.5768
*β*_*s*,*dist*_	−0.1830	−0.1717	−0.0113	0.0042	**0.0124**
*β*_*s*,*dist*^2^_	0.3762	0.3865	−0.0103	0.0121	0.3967
*β*_*s*,*age*×*C*_	0.0784	0.5293	−0.4509	0.3350	0.1783
*β*_*s*,*age*×*Eglob*_	−0.0395	0.6452	−0.6847	0.2648	**0.0150**
*β*_*s*,*age*×*k*_	0.0037	−0.0054	0.0091	0.0277	0.7430
*β*_*s*,*age*×*Q*_	0.1467	−0.6588	0.8055	0.4180	**0.0655**
*β*_*s*,*age*×*l*_	0.0176	0.0464	−0.0288	0.0166	0.0819
*β*_*s*,*age*×*sex*_	−0.0313	−0.0083	−0.0230	0.0215	0.2849

* Adjusted using the adaptive FDR procedure detailed in Benjamini and Hochberg (2000).

a Bold values indicate (marginally) significant changes in the estimated relationships between covariates and network connectivity that occur when shifting from rest to a visual task.

**Table 5.** Aging data: Variance estimates for random effects (excluding propensities and distance random effects) for the rest and visual data fit

**Parameter**	**Variance estimate**			
	**Rest**		**Task**	
	**Young**	**Older**	**Young**	**Older**
*b*_*ri*,0_	0.01388	0.03261	0.02263	0.03586
*b*_*ri*,*C*_	47.30740	29.17890	69.86910	5.86460
*b*_*ri*,*Eglob*_	29.53790	77.37150	117.9700	143.9900
*b*_*ri*,*k*_	0.58320	0.35250	0.75910	0.44390
*b*_*ri*,*l*_	0.11070	0.21230	0.22110	0.17890
*b*_*si*,0_	0.00097	0.00031	0.00095	0.00038
*b*_*si*,*C*_	0.38000	0.54220	0.94000	0.08553
*b*_*si*,*Eglob*_	0.49290	0.13280	0.38670	0.19420
*b*_*si*,*k*_	0.00336	0.00419	0.00663	0.00618
*b*_*si*,*l*_	0.00250	0.00044	0.00239	0.00013

Below we highlight significant population network changes and variability differences (deviations from populations) for rest-visual task pairs gleaned from the last three columns of Table 4 (bolded *p* values) and Table 5.

1. Young adults become more degree assortative (presence and strength) when comparing their visual-state to resting-state networks, whereas older adults do not [Variability: Greater increase in variability (presence and strength) in assortativity for young adults than older adults when comparing their visual-state to resting-state networks].

2. Older adults gain connections (presence) (i.e., have more dense networks) when comparing their visual-state to resting-state networks, whereas younger adults do not [Variability: Older adults have more variability in their density than young adults during both rest and the visual task. However, the variability of young adults increases more than older adults when comparing their visual-state to resting-state networks].

3. Global efficiency (functional integration/distributive processing) plays an even greater role in explaining the connection strength between two regions for younger adults when comparing their visual-state to resting-state networks, whereas it plays less of a role for older adults [Variability: Young adults have more variability in the GE/strength relationship than older adults during both rest and the visual task. The variability in this relationship goes up for older adults when comparing their visual-state to resting-state networks, but goes down for young adults].

4. Brain regions farther apart in distance tend to have relatively weaker connections when comparing visual-state to resting-state networks for both age groups.

5. Young adults have relatively weaker overall connectivity as their brains become more modular when comparing their visual-state to resting-state networks, whereas the converse is true for older adults.

6. There is an overall (across all random effects) larger increase in variability for young adults than older adults when comparing their visual-state to resting-state networks.

**Figure 3.**
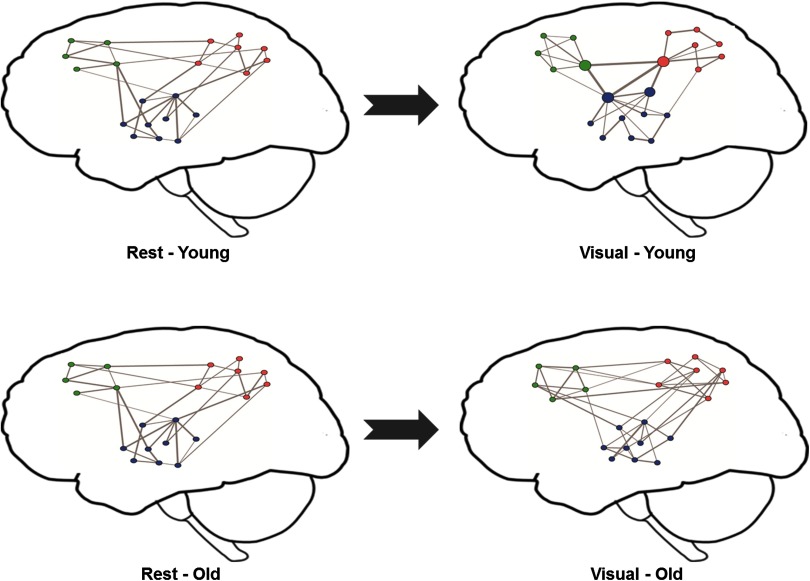
Cartoon depiction of important differences found between the brain networks in young and older adults when comparing their visual-state to resting-state networks. Each network node represents a brain region and the lines represent functional connections. The node color indicates the module membership and the edge thickness represents connection strength (stronger connections are shown with thicker edges). Young adults’ brains shift to a functional architecture comprising a resilient core of interconnected high-degree/high-strength/globally efficient hubs without increasing wiring cost, by minimizing intermodule connectivity, when comparing their visual-state to resting-state networks. This shift does not occur for older adults, and further, their wiring cost increases (i.e., their networks become more densely connected with random connections).

**Figure 4.**
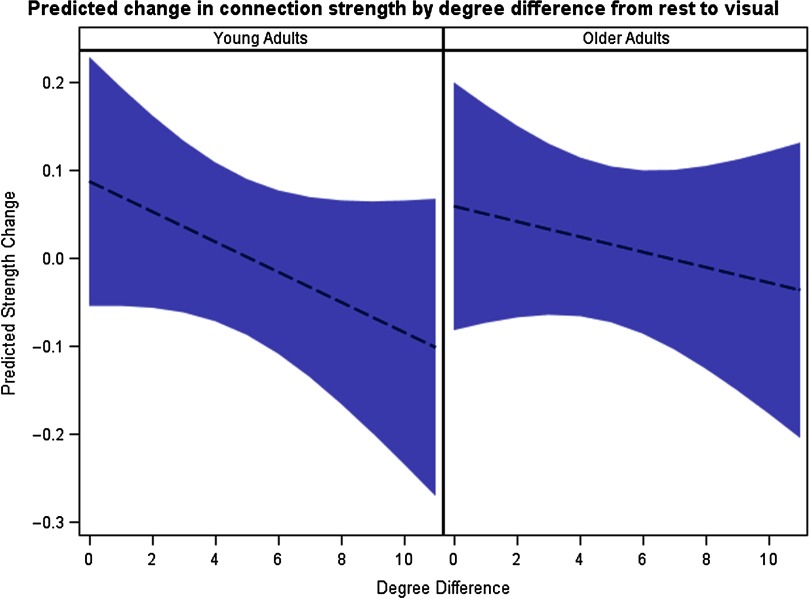
Prediction intervals for rest-to-visual changes in connection strength as a function of degree difference in young and older participants.

Conclusion: Young adults’ brains shift to a functional architecture comprising a resilient core of interconnected high-degree/high-strength/globally efficient hubs (i.e., a “rich-club” organization as discussed in Van Den Heuvel & Sporns, 2011) without increasing wiring cost, by minimizing intermodule connectivity, when comparing their visual-state to resting-state networks. This shift does not occur for older adults, and further, their wiring cost increases (i.e., their networks become more densely connected with random connections). These additional connections may serve to partially compensate for this lack of shifting into tight communities for efficient task performance. The relative lack of a shift towards a resilient core of interconnected high-degree/high-strength/globally efficient hubs suggests that a rest to visual task transition does not strengthen connections within the task-relevant networks as much for older adults. This finding is consistent with cognitive studies showing that older adults are more vulnerable to distraction when performing tasks (Alain & Woods, 1999; Darowski, Helder, Zacks, Hasher, & Hambrick, 2008; Grady, Springer, Hongwanishkul, McIntosh, & Winocur, 2006). These results are visually depicted in Figure 3, which shows two sets of cartoon brain networks that typify the differences found between the brain networks in young and older adults when comparing their visual-state to resting-state networks. Additionally, the degree (strength) assortativity differences are shown in the 95% prediction intervals of Figure 4, which provides a model-based definition of normal ranges for both groups. The predicted strength change is initially higher for young adults and then has a faster decay than for older adults as the disparity between the degrees of two nodes increases, thus implying the noted assortativity differences.

## DISCUSSION

Although network science has catalyzed a paradigmatic shift in neuroscience, the current tools used to analyze network data do not fully harness the wealth of information present, particularly when it comes to multitask analyses. Our multitask mixed-modeling framework fills this void by providing a comprehensive approach to assessing system-level brain properties across multiple tasks, allowing us to uncover relationships between population network differences and individual variability in network differences, in between-task changes, and (health) outcomes of interest in a principled manner. Reanalysis of the aging data from our prior work (Simpson & Laurienti, 2015) illustrated the additional and unique utilities of our approach compared with the unitask mixed-modeling framework (for cross-sectional brain network data), and its use in larger datasets such as those from the Human Connectome Project (Van Essen et al., 2013) will further magnify its appeal. Future analyses will employ our approach with these data to examine the relationship between cognition and rest-to-task brain network changes. For example, much effort has focused on resting brain data to determine differences between those with low and high IQs. However, the underlying mechanism causing IQ differences is likely also related to how the brain changes from one task to another, and not just to average properties during a given task.

Extending our framework to facilitate convergence for data with larger numbers of task states also provides fertile ground for future work. These extensions will also serve to accommodate more spatially resolved networks (hundreds or thousands of nodes) as they will rely on the development of appropriate dimension-reduction methods that can be overlaid on our approach (Bahrami et al., 2018). Another option to facilitate convergence with larger numbers of task states, or with more resolved networks, is to remove the nodal propensity random effects and model the covariance matrix of the remaining random effects with an unstructured matrix to partially compensate for this removal. Incorporating negative connections (i.e., negatively correlated nodes/brain regions) into multitask brain network analyses remains another important goal. The lack of metrics for quantifying functional segregation and integration (e.g., *C* and *Eglob*) with negatively weighted edges currently precludes this.

Our proposed multitask mixed-modeling framework fuses multivariate statistical methods with network-based functional neuroimage analysis to engender a powerful analytic tool for whole-brain multitask network analyses. It fills a critical methodological gap and allows more accurately illuminating neurobiological correlates of brain changes of interest, allowing researchers to investigate how phenotypic traits are related to within-task brain network organization and between-task organizational changes. It enables these investigations at both the group and the individual level, providing a step towards the development of tailored preventive, diagnostic, and treatment strategies to individuals with a variety of brain diseases and disorders.

## FUNDING INFORMATION

Sean Simpson, National Institute of Biomedical Imaging and Bioengineering (http://dx.doi.org/10.13039/100000070), Award ID: K25EB012236. Sean Simpson, National Institute of Biomedical Imaging and Bioengineering (http://dx.doi.org/10.13039/100000070), Award ID: R01EB024559. Sean Simpson, National Center for Advancing Translational Sciences (http://dx.doi.org/10.13039/100006108), Award ID: UL1TR001420. Paul Laurienti, National Institutes of Health (http://dx.doi.org/10.13039/100000002), Award ID: P30 21332.

## AUTHOR CONTRIBUTIONS

Sean Simpson: Conceptualization; Data curation; Formal analysis; Funding acquisition; Investigation; Methodology; Project administration; Resources; Software; Supervision; Validation; Visualization; Writing – original draft. Mohsen Bahrami: Visualization; Writing – original draft. Paul Laurienti: Conceptualization; Data curation; Investigation; Writing – original draft.

## TECHNICAL TERMS

Multivariate modeling: Modeling data with two or more correlated outcome (dependent) variables.
Mixed model: Statistical model containing both fixed (population-level) and random (individual-level) effects used to model multivariate data.
Fixed effects: Variables whose effects are constant across individuals.
Random effects: Variables whose effects vary across individuals.
Euclidean distance: Straight-line distance between two points.
Unstructured (covariance) matrix: A matrix in which no constraints are imposed on the values. Each variance/covariance is estimated uniquely from the data.
Pseudo-likelihood: An approximation to the likelihood function (joint probability distribution) of the observed data for easier computation or estimation.
Bernoulli distribution: Discrete probability distribution for a random variable whose outcome is binary (e.g., can be either 0 or 1).
