# Supplementary material for: A mixed-modeling framework for analyzing multitask whole-brain network data
Source: Netw Neurosci. 2019 Feb 1;3(2):307–24. doi: 10.1162/netn_a_00065 (PMC6370463; doi:10.1162/netn_a_00065)
Supplement: Supplementary file 1 [file netn-03-307-s001.pdf]

Simpson, S. L., Bahrami, M., & Laurienti, P. J. (2019). Supporting information for "A mixed modeling framework for analyzing multitask whole-brain network data."
Network Neuroscience 3(2), 307–324. https://doi.org/10.1162/netn_a_00065

Appendix S1: Macro for model fitting

```
/***************************************************************************/
/* The syntax for the macro is:                                            */
/*                                                                         */
/* %mixnet (data= ,response= ,COIcovars= ,CONcovars= ,NETcovars= ,         */
/*                            INTcovars,Bin_REs,Cont_REs,subject),        */
/*                                                                         */
/*                                                                         */
/*        "data"      specifies the dataset to be used                     */
/*        "response"  specifies the Y (outcome) variable                   */
/*        "COIcovars" list of the covariates of interest                   */
/*        "CONcovars" list of the confounding covariates                   */
/*        "NETcovars" list of the network covariates                       */
/*        "INTcovars" list of the interaction covariates                   */
/*        "Bin_REs  " list of random effects statements for Part I model   */
/*        "Cont_REs"  list of random effects statements for Part II model */
/*        "subject"   variable that indicates the subject id               */
/*                                                                         */
/***************************************************************************/

%macro mixnet(data,response,COIcovars,CONcovars,NETcovars,INTcovars,Bin_REs,
             Cont_REs,subject);

data data_b;
 set &data;
 if &response=0 then YN=0;
 else if &response>0 then YN=1;
run;

data data_n;
 set &data;
 if &response>0;
run;

proc sort data=data_b; by &subject; run;
proc sort data=data_n; by &subject; run;

/*Binomial Model*/
%hpglimmix(data=data_b,
  stmts=%str(class &COIcovars &subject;
           model yn = &COIcovars &CONcovars &NETcovars &INTcovars/solution;
           &Bin_REs; ods output covparms=covpb;),error=binary)
run;

data covpb_new;
 set covpb;
 if covparm ne "Residual";
run;

proc glimmix data = data_b;
 class &COIcovars (ref=first);
 model yn (desc)= &COIcovars &CONcovars &NETcovars &INTcovars
  /dist=binary ddfm=residual solution;
 &Bin_REs;
 parms/pdata=covpb_new noiter;
run;
```

```
/*Normal Model*/
proc hpmixed data = data_n noclprint;
 class &COIcovars;
 model n_aij = &COIcovars &CONcovars &NETcovars &INTcovars /solution;
 &Cont_REs;
 ods output covparms=covpn;
run;

proc glimmix data = data_n noprofile;
 class &COIcovars (ref=first);
 model n_aij = &COIcovars &CONcovars &NETcovars &INTcovars
  /dist=n ddfm=residual solution;
 &Cont_REs;
 parms/pdata=covpn_new noiter;
run;

%mend;
```

Defining Random Effects Statements (example for Rest/Visual Model)

```
%let Bin_RE1 = int visual;
%let Cont_RE1 = int visual;
%let Bin_RE2 = cent_clust_avg visual*cent_clust_avg;
%let Cont_RE2 = cent_clust_avg visual*cent_clust_avg;
%let Bin_RE3 = cent_eglob_avg visual*cent_eglob_avg;
%let Cont_RE3 = cent_eglob_avg visual*cent_eglob_avg;
%let Bin_RE4 = cent_deg_diff visual*cent_deg_diff;
%let Cont_RE4 = cent_deg_diff visual*cent_deg_diff;
%let Bin_RE5 = cent_lev_avg visual*cent_lev_avg;
%let Cont_RE5 = cent_lev_avg visual*cent_lev_avg;
%let Bin_RE6 = cent_distance visual*cent_distance;
%let Cont_RE6 = cent_distance visual*cent_distance;
%let Bin_RE7 = cent_distance_2 visual*cent_distance_2;
%let Cont_RE7 = cent_distance_2 visual*cent_distance_2;

*Random Effects Statements;
%macro RandomEffectsStatements(NumberOfNodes, StatementNumber);
 %do i = 1 %to & NumberOfNodes;
   %let j = %eval(&i+7);
   %let Bin_RE&j = NodeInd_&i visual*NodeInd_&i;
   %let Cont_RE&j = NodeInd_&i visual*NodeInd_&i;
 %end;
 %do i = 1 %to &StatementNumber;
   %let Bin_REState&i = random &Bin_RE&i/
        sub=%str(&subject) type=chol group=%str(&COIcovars;);
   %let Cont_REState&i = random &Cont_RE&i/
       sub=%str(&subject) type=chol group=%str(&COIcovars;);
%end;
```
