# Supplementary material for: A mixed-modeling framework for analyzing multitask whole-brain network data
Source: Netw Neurosci. 2019 Feb 1;3(2):307–24. doi: 10.1162/netn_a_00065 (PMC6370463; doi:10.1162/netn_a_00065)
Supplement: Supplementary file 2 [file netn-03-307-s002.pdf]

Simpson, S. L., Bahrami, M., & Laurienti, P. J. (2019). Supporting information for "A mixed modeling framework for analyzing multitask whole-brain network data." *Network Neuroscience*, *3*(2), 307–324. https://doi.org/10.1162/netn_a_00065

1. More accurate and precise within-task results

1. Clustering coefficient (functional segregation/regional specificity) is shown to play an even greater role in explaining the connectivity (presence) between two regions at rest for both young and older adults as indicated by the change in the order of magnitude of $\beta_{rl_1,C}$, the increase in $(\beta_{rl_1,C} + \beta_{rl_1,age\times C})$, and the change in two orders of magnitude of the p-value for $\beta_{r,C}$. Additionally, the change in sign of $\beta_{rl_1,age\times C}$ provides (weak) evidence that older adults have a weaker relationship between clustering and connectivity than young adults, whereas the opposite conclusion results from the unitask model fit.

2. The change in sign of $\beta_{rl_1,Q}$ and $\beta_{rl_1,age\times Q}$ provides (weak) evidence that the relationship between modularity and connectivity is actually the inverse of the one estimated by the unitask model for both young and older adults.

3. The change in sign of $\beta_{rl_1,age\times l}$ provides (weak) evidence that older adults have a weaker relationship between leverage centrality and connectivity than young adults, whereas the opposite conclusion results from the unitask model fit.

4. Modularity is shown to have an even stronger negative relationship with connection strength for young adults as evidenced by the change in the order of magnitude of $\beta_{sl_1,Q}$ and the 81% reduction in its associated p-value. Additionally, older adults are no longer estimated to have a stronger negative relationship between modularity and connection strength than young adults as indicated by the two orders of magnitude increase in the p-value associated with $\beta_{sl_1,age\times Q}$.

5. The change in the p-value associated with $\beta_{sl_1,age\times C}$ from significant to non-significant implies that there **is not** evidence of a different relationship between clustering and connection strength for older adults than young adults at rest as concluded from the unitask model fit.

6. The change in sign of $\beta_{sl_1,age\times k}$ provides (weak) evidence that the brain networks of older adults are actually more degree assortative (in terms of connection strength) than young adults at rest, not less as the unitask model indicates.

Supplementary Table 1. Aging data: estimates, standard errors (se), and p-values for the original univariate mixed model fit to Rest data and new multivariate fit to Rest (and Multisensory) data.

Parameter	Rest (Univariate)			[a]Rest (with MS)		
l_1 =rest	Estimate	SE	*P-value	Estimate	SE	*P-value
$\beta_{rl_1,0}$	-0.3141	0.0569	< 0.0001	-0.2614	0.0467	< 0.0001
$\beta_{rl_1,C}$	0.7807	0.3424	0.0355	***7.2829***	1.7689	***0.0001***
$\beta_{rl_1,Eglob}$	32.6231	2.3322	< 0.0001	30.8250	1.4366	< 0.0001
$\beta_{rl_1,k}$	-1.4442	0.1522	< 0.0001	-1.5301	0.1748	< 0.0001
$\beta_{rl_1,Q}$	-0.7345	1.1361	0.5179	***0.1268***	0.9497	0.8938
$\beta_{rl_1,l}$	1.1598	0.0785	< 0.0001	1.3945	0.0861	< 0.0001
$\beta_{rl_1,age}$	-0.0438	0.0773	0.5709	-0.0906	0.0779	0.2568
$\beta_{rl_1,sex}$	-0.0085	0.0825	0.9178	-0.0147	0.0686	0.8304
$\beta_{rl_1,educ}$	0.0027	0.0103	0.7954	0.0032	0.0098	0.7437
$\beta_{rl_1,dist}$	-1.4266	0.0572	< 0.0001	-1.4582	0.0517	< 0.0001
$\beta_{rl_1,dist^2}$	2.6558	0.1417	< 0.0001	2.6559	0.1147	< 0.0001
$\beta_{rl_1,age \times C}$	1.1249	0.7986	0.1943	***-1.8954***	2.3517	0.4203
$\beta_{rl_1,age \times Eglob}$	-1.7255	3.3478	0.6063	-0.4546	2.6167	0.8621
$\beta_{rl_1,age \times k}$	0.2455	0.2185	0.2873	0.2536	0.2253	0.2603
$\beta_{rl_1,age \times Q}$	1.5858	1.5753	0.3141	***-0.9548***	1.4523	0.5109
$\beta_{rl_1,age \times l}$	0.0638	0.1154	0.5803	***-0.0736***	0.1438	0.6088
$\beta_{rl_1,age \times sex}$	0.1914	0.1145	0.1301	0.2173	0.1164	0.0814
$\beta_{sl_1,0}$	0.2290	0.0091	< 0.0001	0.2317	0.01106	< 0.0001
$\beta_{sl_1,C}$	2.2940	0.2428	< 0.0001	2.2510	0.1776	< 0.0001
$\beta_{sl_1,Eglob}$	0.9534	0.1823	< 0.0001	1.0508	0.1833	< 0.0001
$\beta_{sl_1,k}$	-0.2524	0.0153	< 0.0001	-0.2445	0.0139	< 0.0001
$\beta_{sl_1,Q}$	-0.0373	0.1723	0.8285	***-0.2990***	0.1611	***0.1613***
$\beta_{sl_1,l}$	-0.0036	0.0109	0.7426	-0.0039	0.0124	0.7550

Supplementary Table 2. Aging data (multivariate Rest/MS fit): estimates for Multisensory and Rest, and estimates, standard errors (se), and p-values for the between-task differences.

	MS	Rest	[a]Difference (MS - Rest)		
Parameter	Estimate	Estimate	Estimate	SE	*P-value
$\beta_{r,0}$	-0.0813	-0.2614	0.1801	0.0842	**0.0588**
$\beta_{r,C}$	13.2286	7.2829	5.9457	1.5744	**0.0004**
$\beta_{r,Eglob}$	34.537	30.8250	3.7120	2.5545	0.1706
$\beta_{r,k}$	-2.2216	-1.5301	-0.6915	0.2384	**0.0078**
$\beta_{r,Q}$	-3.2427	0.1268	-3.3695	2.2266	0.1608
$\beta_{r,l}$	1.5428	1.3945	0.1483	0.0723	**0.0648**
$\beta_{r,age}$	0.0098	-0.0906	0.1004	0.1172	0.3918
$\beta_{r,sex}$	-0.0326	-0.0147	-0.0179	0.1311	0.8912
$\beta_{r,educ}$	0.0189	0.0032	0.0157	0.0174	0.3676
$\beta_{r,dist}$	-1.5237	-1.4582	-0.0655	0.0616	0.2878
$\beta_{r,dist^2}$	3.0478	2.6559	0.3919	0.1977	**0.0664**
$\beta_{r,age\times C}$	-9.3597	-1.8954	-7.4643	2.4526	**0.0055**
$\beta_{r,age\times Eglob}$	-3.3786	-0.4546	-2.9240	3.0551	0.3385
$\beta_{r,age\times k}$	0.8434	0.2536	0.5898	0.2776	**0.0588**
$\beta_{r,age\times Q}$	4.9509	-0.9548	5.9057	2.9792	**0.0664**
$\beta_{r,age\times l}$	-0.2156	-0.0736	-0.1420	0.1083	0.2096
$\beta_{r,age\times sex}$	0.1027	0.2173	-0.1146	0.1841	0.5336
$\beta_{s,0}$	0.2271	0.2317	-0.0046	0.0118	0.7111
$\beta_{s,C}$	1.9784	2.2510	-0.2726	0.2313	0.4454
$\beta_{s,Eglob}$	1.2290	1.0508	0.1782	0.2376	0.6347
$\beta_{s,k}$	-0.2645	-0.2445	-0.0200	0.0141	0.3662
$\beta_{s,Q}$	-0.2540	-0.2990	0.0450	0.3275	0.8907
$\beta_{s,l}$	-0.0108	-0.0039	-0.0069	0.0124	0.7034

Supplementary Table 3. Aging data: variance estimates for random effects (excluding propensities) for the Rest and Multisensory data fit.

	Variance Estimate			
	Rest		Task	
Parameter	Young	Older	Young	Older
$\boldsymbol{b}_{ri,0}$	0.01388	0.03261	0.05121	0.05565
$\boldsymbol{b}_{ri,C}$	47.30740	29.17890	1.24400	27.14320
$\boldsymbol{b}_{ri,Eglob}$	29.53790	77.37150	99.67160	22.66260
$\boldsymbol{b}_{ri,k}$	0.58320	0.35250	1.06640	0.31590
$\boldsymbol{b}_{ri,l}$	0.11070	0.21230	0.01208	0.02208
$\boldsymbol{b}_{si,0}$	0.00097	0.00031	0.00102	0.00071
$\boldsymbol{b}_{si,C}$	0.38000	0.54220	0.29610	0.00471
$\boldsymbol{b}_{si,Eglob}$	0.49290	0.13280	0.68300	0.06874
$\boldsymbol{b}_{si,k}$	0.00336	0.00419	0.00281	0.00488
$\boldsymbol{b}_{si,l}$	0.00250	0.00044	0.00169	0.00014

2. Assess population network differences and individual variability in network differences within and between tasks

Below we highlight significant population network changes and variability differences (deviations from populations) for rest-multisensory task pairs gleaned from the last three columns of Supplementary Table 2 (bolded p-values) and Supplementary Table 3.

1. Young and older adults gain connections (presence) (i.e., have more dense networks) when comparing their multisensory-state to resting-state networks [Variability: Older adults have more variability in their density than young adults during both rest and the multisensory task. However, the variability of young adults increases more than older adults when comparing their multisensory-state to resting-state networks].

2. Clustering (functional segregation/regional specificity) plays an even greater role in explaining the connectivity (presence) between two regions for young adults when comparing their multisensory-state to resting-state networks, whereas it plays less of a role for older adults [Variability: Young adults have more variability in the clustering/presence relationship than older adults at rest, but this variability drops by more than an order of magnitude when comparing their multisensory-state to resting-state networks, whereas the variability of older adults remains essentially the same.].

3. Young adults become more degree assortative (presence) when comparing their multisensory-state to resting-state networks, whereas older adults do not [Variability: Greater increase in variability (presence) in assortativity for young adults than older adults when comparing their multisensory-state to resting-state networks (variability of older adults actually decreases)].

4. Leverage centrality plays more of a role in explaining the connectivity (presence) between two regions for young adults when comparing their multisensory-state to resting-state networks, whereas it does not for older adults [Variability: Older adults have more variability in the LC/presence relationship than young adults during both rest and the multisensory task. The variability in this relationship drops by an order of magnitude when comparing multisensory-state to resting-state networks for both older and young adults.].

5. Older adults have relatively denser networks as their brains become more modular when comparing their multisensory-state to resting-state networks, whereas young adults do not.

- To clarify, this result does not mean that older adult brains become more modular than young adult brains when comparing their multisensory-state to resting-state networks, just that at the same level of modularity older adults have denser networks and become denser at a faster rate as their modularity increases. That is, for older adults, becoming more modular comes at the expense of a relative increase in wiring cost, a cost that young adults do not incur.

6. Brain regions farther apart in distance tend to have relatively weaker connections when comparing multisensory-state to resting-state networks for both age groups.

7. Older adults have an overall (across all random effects) decrease in variability when comparing their multisensory-state to resting-state networks, whereas young adults have an increase in variability.

Conclusion: Similar (though not identical) to what was observed when comparing visual to rest, young adults' brains shift to a functional architecture comprising a resilient core of interconnected high-degree/locally efficient hubs when comparing their multisensory-state to resting-state networks, but wiring cost is increased some to accomplish this, likely due to the additional inter-module connectivity needed for a multisensory task (as opposed to a unisensory visual task). This shift does not occur for older adults, but they do also experience an increase in wiring cost (i.e., their networks become more densely connected with random connections). The relative lack of a shift towards a resilient core of interconnected high-degree/locally efficient hubs suggests that a rest to multisensory task transition does not increase the connectivity within the task-relevant networks as much for older adults. This finding is again consistent with the cognitive studies showing the vulnerability of older adults to distraction when performing tasks. These results are visually depicted in Supplementary Figure 1 which shows two sets of cartoon brain networks that illustrate the differences found between the brain networks in young and older adults when comparing their multisensory-state to resting-state networks. Additionally, the degree (strength) assortativity differences are shown in the 95% prediction intervals of Supplementary Figure 2. While the differences between the two groups were not significant, the predicted strength change is initially higher for young adults and then has a faster decay than for older adults as the disparity between the degrees of two nodes increases, thus implying a trend towards assortativity differences.

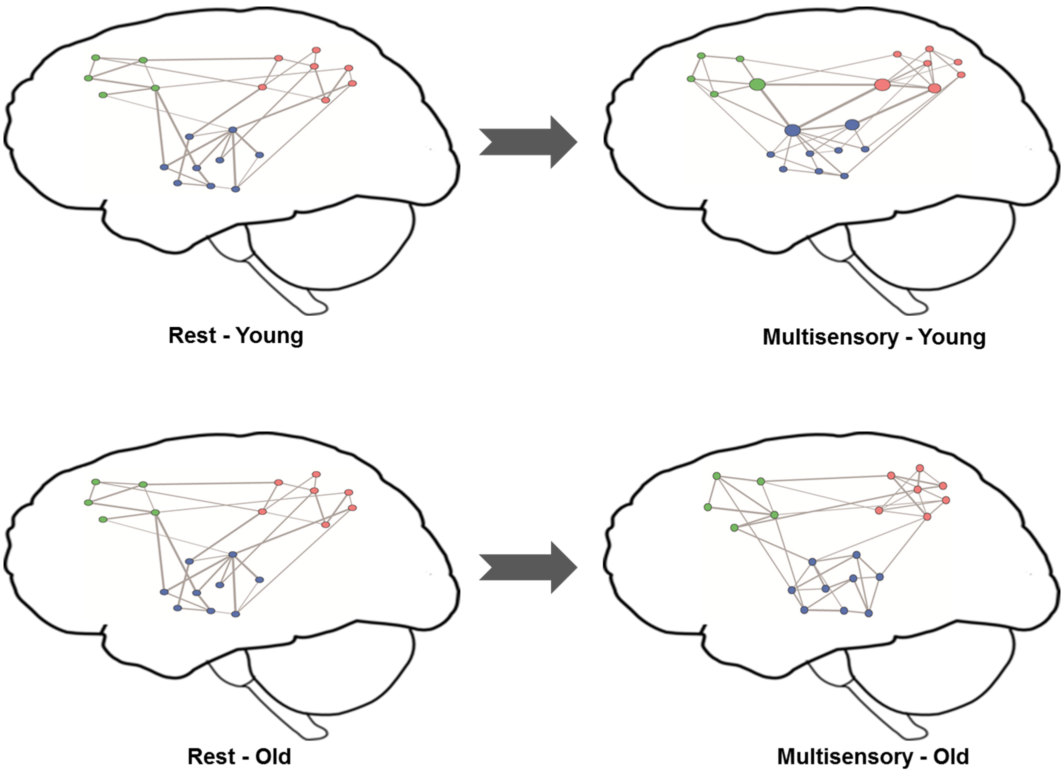

Supplementary Figure 1. Cartoon depiction of important differences found between the brain networks in young and older adults when comparing their multisensory-state to resting-state networks. Each network node represents a brain region and the lines represent functional connections. The node color indicates the module membership and the edge thickness represents connection strength (stronger connections are shown with thicker edges). Young adults' brains shift to a functional architecture comprising a resilient core of interconnected high-degree/locally efficient hubs when comparing their multisensory-state to resting-state networks, but wiring cost is increased some to accomplish this. This shift does not occur for older adults, but they do also experience an increase in wiring cost (i.e., their networks become more densely connected with random connections).

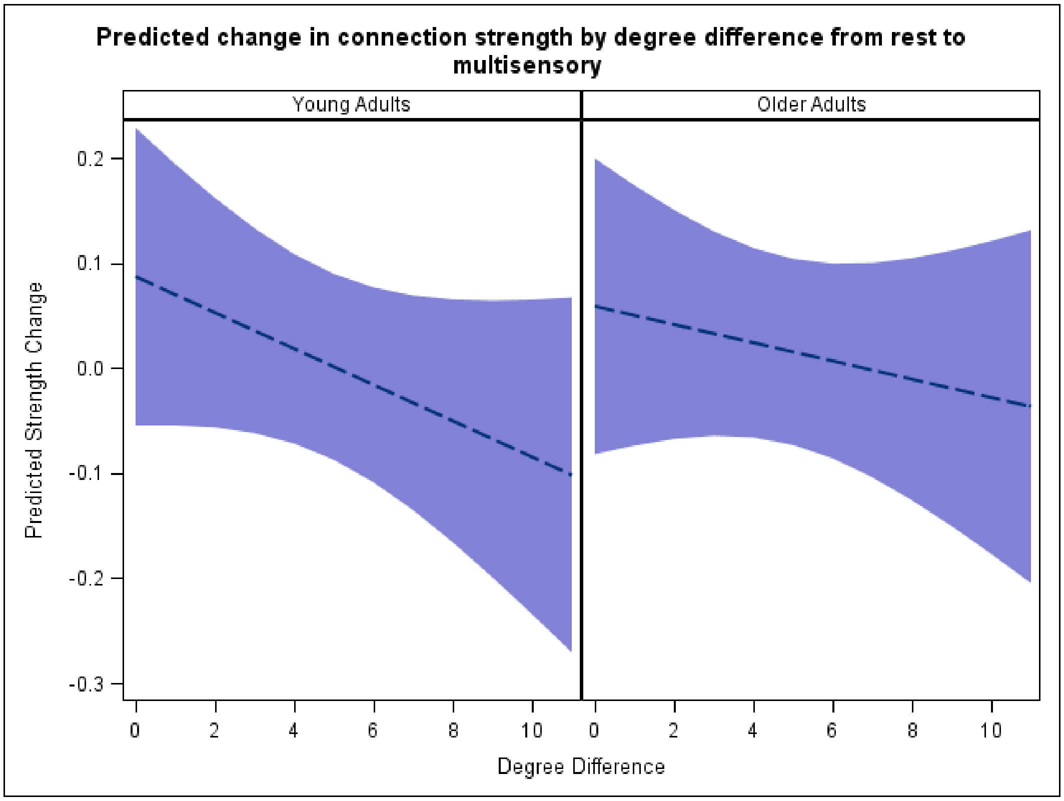

90 **Supplementary Figure 2.** Prediction intervals for rest-to-multisensory changes in connection strength as a function of degree difference in young and older

91 participants.
